# Circulating microRNAs from the mouse tibia fracture model reflect the signature from patients with complex regional pain syndrome

**DOI:** 10.1097/PR9.0000000000000950

**Published:** 2021-08-24

**Authors:** Jason R. Wickman, Xuan Luo, Wenwu Li, Renee Jean-Toussaint, Peyman Sahbaie, Ahmet Sacan, J. David Clark, Seena K. Ajit

**Affiliations:** a Pharmacology and Physiology, Drexel University College of Medicine, Philadelphia, PA, USA; b Anesthesiology Service, Veterans Affairs Palo Alto Health Care System, Palo Alto, CA, USA; c School of Biomedical Engineering, Science and Health Systems, Drexel University, Philadelphia, PA, USA; d Department of Anesthesiology, Perioperative, and Pain Medicine, Stanford University School of Medicine, Stanford, CA, USA

**Keywords:** Complex regional pain syndrome, Tibia fracture model, miRNA, Extracellular vesicles, Exosomes, Biomarker

## Abstract

**Introduction:**

Complex regional pain syndrome (CRPS) often results from an initial trauma that later produces a disproportionate amount of pain. The mechanisms underlying CRPS have been studied using a tibia fracture model (TFM) in rodents because this model closely mimics symptoms and has several molecular correlates observed in patients with CRPS.

**Objective:**

Here, we determined whether the TFM has alterations in circulating microRNAs (miRNAs) and cytokines transported by small extracellular vesicles (sEVs) that faithfully model previously reported miRNA alterations from patients with CRPS.

**Methods:**

We isolated and characterized serum-derived sEVs from mice 3 weeks after fracture when symptoms such as pain hypersensitivity develop. Whole-transcriptome profiling was used to determine sEV miRNAs, and Bio-Plex Pro Mouse Cytokine 23-plex assay was used to measure cytokines. Differentially expressed miRNAs from TFM were compared with previously reported circulating miRNA alterations from patients with CRPS.

**Results:**

Although sEV cytokine levels were unchanged, there were significant changes in sEV miRNA profiles. Differentially expressed miRNAs from TFM sEVs significantly overlapped with those previously reported in patients with CRPS. Of the 57 sEV miRNAs dysregulated in the TFM, 30 were previously reported in patients with CRPS compared with healthy control donors both in sEVs and 23 in whole blood.

**Conclusions:**

These findings enhance the validity of TFM as a model for CRPS and suggest that specific miRNA dysregulation may be a shared feature of CRPS and the TFM. These dysregulated miRNAs could help identify mechanistic targets or serve as biomarker candidates for both diagnosis and treatment responses in clinical trials.

## Abstract 2

Supplemental Digital Content is Available in the Text.

Whole-transcriptome profiling of circulating microRNA from the mouse tibia fracture model enabled the identification of commonly dysregulated microRNA previously reported in patients with complex regional pain syndrome.

## 1. Introduction

Complex regional pain syndrome (CRPS) is a chronic disease where patients experience intense disabling pain, motor, and autonomic dysfunction that can significantly decrease the quality of life.^7,10^ Complex regional pain syndrome is most commonly incited by an acute trauma to a limb such as a fracture^11,23,32^ and diagnosed with or without attributable peripheral nerve injury as CRPS I or CRPS II, respectively, and is more prevalent in women than men (3:1).^10,19,38^ The etiology and pathology of CRPS are still poorly understood, and there is no quantitative method to stratify these populations.^28^ Aberrant local and systemic cytokine signaling in patients with CRPS are commonly observed.^35^ Although these are promising biomarkers of inflammation, the multifactorial nature of CRPS and patient heterogeneity further hampers their utility in early diagnosis and intervention.^28^ Clinical biomarkers with increased specificity for patients with CRPS would have a significant impact on better understanding disease mechanisms, diagnosing patient populations, predicting patient outcomes, and identifying effective treatment strategies.^4,5^ As no disease-modifying treatments are available, preclinical animal models must robustly mimic disease etiology and progression to ensure impactful translational research for therapeutic and diagnostic development.

The rodent tibia fracture model (TFM) closely mimics the symptoms of patients with CRPS, including mechanical allodynia and hyperalgesia, limb edema, motor dysfunction, keratinocyte proliferation, autoantibody generation, alterations in neuropeptide signaling, and decreases in memory; increase in peripheral inflammatory mediators was more pronounced in the acute phase.^6^ However, there are no reports that investigate how microRNA (miRNA) signatures in patients with CRPS compared with the TFM. MicroRNAs are small noncoding RNAs that can negatively regulate gene expression, and we have previously reported the potential utility of circulating miRNAs in whole blood and serum-derived small extracellular vesicles (sEVs) as diagnostic, therapeutic, and mechanistic markers for CRPS.^15,34,36,39^ Better understanding of miRNA dysregulation in the TFM would enhance the face validity of the model, enable mechanistic studies with relevance to CRPS, and help identify key regulators of disease progression.

Here, we examined the miRNA signatures of serum-derived sEVs from male TFM and control mice and compared them with reported miRNAs dysregulated in patients with CRPS (male and female) to examine underlying common alterations. sEVs are 30 to 150 nm vesicles of endosomal origin and can transport selectively packaged cargo from the cell of origin across long distances within the body.^50^ Disease pathology can alter the miRNAs packaged into sEVs, potentially demonstrating a superior molecular signature of disease compared with circulating miRNAs in blood or serum.^12,45^ We hypothesized that dysregulated miRNAs in sEVs from TFM mice would overlap with those observed in patients with CRPS. To test this, we characterized sEVs from TFM mice at 3 weeks after injury, where pain hypersensitivity is established. We analyzed both miRNAs and cytokines from serum-derived sEVs to identify the differential expression in TFM sEVs compared with controls. We also compared differentially expressed sEV miRNAs with previously identified miRNAs from patients with CRPS.

## 2. Materials and methods

### 2.1. Experimental animals

These experiments were approved by the Veterans Affairs Palo Alto Health Care System's Institutional Animal Care and Use Committee (Palo Alto, CA) and followed the animal subject guidelines of the International Association for the Study of Pain.

Male C57BL/6J mice aged 12 to 14 weeks (Jackson Laboratory, Bar Harbor, ME) were housed in groups of 4 (12-hour light or dark cycle, ambient temperature 22 ± 3°C, and with food and water available ad libitum). After fracture surgery, mice were housed in isolator cages with solid floors covered in 3 cm of soft bedding with access to food and water ad libitum.

### 2.2. Tibia fracture model

Surgery was performed under isoflurane anesthesia, and a closed fracture just distal of the middle of the right tibia was made using a hemostat. The fractured hind limb was wrapped in casting tape (Delta-Lite) such that the hip, knee, and ankle were all fixed. The cast extended from the metatarsals of the hind paw up to a spica formed around the abdomen. A window was left open of the dorsal paw and ankle to prevent constriction when postfracture edema developed.^14,43^ Control mice did not receive any surgery.

### 2.3. Hind paw nociceptive testing

Mechanical allodynia was measured by the up–down von Frey method and hind paw unweighting determined with an incapacitance device (IITC, Woodland Hills, CA) as previously described.^41^ In brief, von Frey fibers were applied to the hind paw plantar skin and withdrawal thresholds estimated by data-fitting algorithm to allow for parametric analysis. Hind paw unweighting was measured with 6 consecutive 6-second measurements at 10-second intervals and data analyzed as a ratio of weight distribution ([2R/(R + L)] × 100%).

### 2.4. Serum collection

Three weeks after tibia fracture or sham procedure, TFM and control mice were placed under isoflurane anesthesia and blood was collected through cardiac puncture. After clotting at room temperature, blood was centrifuged at 10,000*g*. Serum stored at −80°C was shipped to Drexel University for further analysis.

### 2.5. Small extracellular vesicles isolation from serum

Sera from TFM and control mice were processed in parallel as described previously.^22,31^ Five hundred microliters of serum per mouse was diluted with an equal volume of phosphate buffered saline (PBS) and centrifuged for 30 minutes at 2,000*g* at 4°C. RNase inhibitors were added at 1 U/mL (Promega, Madison, WI; RNAsin Plus) to the supernatant and at all subsequent steps at 1 U/µL. Samples were centrifuged for 45 minutes at 12,000*g*, 4°C; supernatant filtered through a 0.22 μm filter; and followed by ultracentrifugation for 2 hours at 110,000*g* at 4°C. Small extracellular vesicle deplete fraction and pellet containing sEVs were washed using PBS and centrifuged at 110,000*g* for 70 minutes at 4°C. sEV pellet was collected after repeating the wash step, which was then resuspended in 100 μL PBS and stored at −80°C.

### 2.6. Nanoparticle tracking analysis

Small extracellular vesicle samples were analyzed for concentration and size using the NanoSight LM10-HS according to the manufacturer's protocol (Malvern Panalytical Inc, Westborough, MA). In brief, samples were diluted 100× in 0.22-μm filtered PBS and continuously injected with a syringe pump. Five measurements in the 488 nm channel were taken for 60 seconds each per sample. Analysis was performed using the nanoparticle tracking analysis (NTA) 3.4.003 software, and the particle concentration and size were taken as the mean of the 5 replicate measurements.

### 2.7. Transmission electron microscopy

Small extracellular vesicle samples were diluted 2× in 4% paraformaldehyde made in 0.2M Sorenson Phosphate Buffer (PB). Ten μL of the sample was pipetted onto parafilm and adsorbed onto either formvar or silicone monoxide copper (EMS) or formvar or carbon nickel (Electron Microscopy Sciences, [EMS], Hatfield, PA) grids for native or immunolabeling, respectively. Grids were washed thrice in 0.1M PB for 2 minutes each, followed by fixation in 1% glutaraldehyde in 0.1M PB for 5 minutes. Grids were washed in distilled water 8 times for 2 minutes each and then stained for contrast in 1% uranyl acetate (EMS) for 2 minutes and embedded in 0.2% uranyl acetate (in 1.6% methyl cellulose) for 10 minutes at 4°C. Excess solution was removed by Whatman filter paper and grids dried. Alternatively, grids were blocked in 5% bovine serum albumin (in 0.1M PB) and then immunolabeled with CD81 (Santa Cruz, Dallas, TX, sc-166209) or CD9 (Santa Cruz, sc-13118) primary antibody (1:100) followed by 10 nm gold-conjugated secondary antibody (1:100) (EMS, 25815) and subsequent contrast and embedding. Images were acquired using a FEI Tecnai 12 120 keV digital transmission electron microscopy equipped with an AMT XR111 CCD camera.

### 2.8. Western blotting

Small extracellular vesicles or sEV deplete samples were diluted 2× in lysis buffer (ThermoFisher, Waltham, MA). For cell lysate, Jurkat cells (ATCC, Gaithersburg, MD, TIB-152) were resuspended in lysis buffer. Five µg lysate was loaded on a 12% Tris-Glycine gel (Invitrogen, Waltham, MA) and resolved under SDS-PAGE. Gel was transferred to the PVDF membrane and the membrane blocked using Intercept Blocking Buffer (Li-Cor, Lincoln, NE). Blots were probed for Calnexin (Abcam, Waltham, MA, ab10286), Glyceraldehyde 3-phosphate dehydrogenase (GAPDH) (CST, Danvers, MA, 2118S), Albumin (Proteintech, Rosemont, IL, 16475-1-AP), HSP70 (Santa Cruz, sc-32239), or CD81 (Santa Cruz, sc-166029) overnight at 4°C (1:500 in tris-buffered saline with tween (TBST)-10% blocking buffer). Blots were washed thrice in TBST and then probed with either donkey anti-rabbit or goat anti-mouse secondary horseradish peroxidase (HRP) antibodies (Abcam, ab16284, ab6789). Blots were developed with supersignal substrate (ThermoFisher) and imaged using the Odyssey FC imager (Li-Cor) or FluorChem M system (ProteinSimple, Santa Clara, CA).

### 2.9. Cytokine profiling

Small extracellular vesicle deplete samples were centrifuged at 10,000*g* for 10 minutes at 4°C. Small extracellular vesicles and sEV deplete samples were diluted 2× in lysis buffer. Protein concentration was measured using a DC Protein assay. sEVs or sEV deplete samples were assayed in duplicate (2× and 4×, respectively) using a mouse 23-Plex cytokine Bio-Plex Pro assay (Bio-Rad, Hercules, CA M60009RDPD) according to the manufacturer's instructions and imaged with the Luminex 100 xMAP system.

### 2.10. Statistical analysis

Group differences in sEV characteristics and total protein content were compared by the 2-tailed Student *t* test, where a *P* value of <0.05 was considered significant. Differences between groups in cytokine content were compared using a 2-tailed Welch *t* test, with Benjamini–Hochberg correction for multiple comparisons at a false discover rate (FDR) of <0.05. Mechanical sensitivity and weight-bearing data were analyzed by unpaired Student *t* tests, and differences are considered significant at a *P* value < 0.05. All data are represented as mean ± SEM except where otherwise noted, and 95% confidence interval is used alternatively. GraphPad Prism version 9.0.0 was used for statistical analysis.

### 2.11. Whole-transcriptome microRNA profiling

Tibia fracture model and control sEV samples were diluted 2× with HTG Biofluids Lysis buffer (HTG Molecular Diagnostics, Inc., Tucson, AZ) to a concentration of ∼10^10^ to 10^11^ sEVs/mL and stored at −80°C. Samples were processed at HTG Molecular Diagnostics in a nuclease-protected miRNA whole-transcriptome assay which allows for the quantitative detection of 2,083 miRNA transcripts. Thirty μL of the sample was used and assayed according to manufacturer protocols. Three samples containing human brain RNA were used as intrarun controls. Samples that did not have ≥10^6^ total read counts, with ≥10% of total reads from a positive spiked in the control, or showed a coefficient of variance ≥15% were removed for low quality. Thus, control 1 was removed because >10% of total reads were from the positive-spiked control. The remaining samples were used for the presence–absence analysis and differential expression analysis. Reads per gene were normalized to transcripts per kilobase million (TPM). Transcripts per kilobase million values were log2 transformed. Low-expressed miRNAs were removed from further analysis if all replicates in both control and TFM groups had TPM values <1. For the presence–absence analysis, a miRNA was considered present in a group if it had a TPM value of ≥1 in every replicate of that group. To compare the samples in their overall miRNA expression levels, a hierarchical clustergram of the samples using the Pearson correlation coefficient was generated. Comparison between TFM and control groups was performed using a permutation *t* test,^21^ and significantly, differentially expressed genes were defined as having a Type I error *P* value of <0.01 and an absolute fold change ≥ 2. Sequencing data are available under the BioProject ID: PRJNA729070.

### 2.12. MicroRNA target gene enrichment analysis

Gene targets of differentially expressed miRNAs were identified using computationally predicted targets from TargetScan^1^ (aggregate P_CT_ > 0.9) and targets with experimental evidence from miRTarBase;^13^ the targets shared between these 2 databases were used to generate a list of common gene targets. ShinyGo V0.60 was used to conduct a hierarchical clustering and enrichment analysis of the common target genes according to Gene Ontology (GO) biological process terms,^17^ using a false discover rate cutoff of 0.05.

## 3. Results

### 3.1. Pain hypersensitivity of tibia fracture model mice

**Figure 1.**
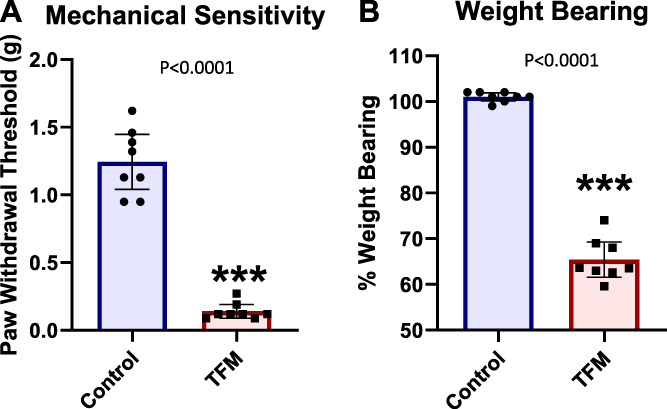
Pain hypersensitivity of TFM and control mice. Mechanical allodynia was measured using von Frey filaments (A), and hind paw unweighting was assessed using an incapacitance device (B) using TFM (n = 8) and control (n = 8) mice 3 weeks postfracture surgery when casts were removed. Mechanical sensitivity and weight-bearing data were analyzed by unpaired Student *t* tests. All data are presented as the mean ± SEM, and differences are considered significant at a *P* value <0.05. TFM, tibia fracture model.

Three weeks after fracture, when casts were removed, control and TFM mice were tested for hind paw mechanical sensitivity and weight bearing. Tibia fracture model mice showed a significantly decreased paw withdrawal threshold of the ipsilateral hind limb and significantly decreased weight bearing (Figs. 1A–B).

### 3.2. Small extracellular vesicle isolation and characterization

**Figure 2.**
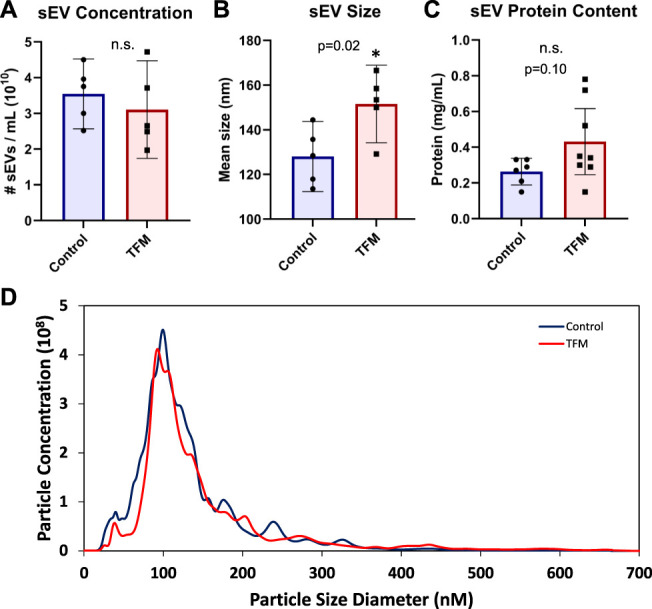
Characterization of serum-derived sEVs of TFM and control mice. Nanoparticle tracking analysis was performed on sEVs isolated form TFM (n = 5) or control (n = 5) mice to measure their concentration (A) and size (B). Protein content of TFM (n = 8) and control (n = 6) sEVs as measured by DC protein assay (C). Average distribution of sEVs from TFM and control as measured by NTA (D). NTA, nanoparticle tracking analysis; TFM, tibia fracture model.

**Figure 3.**
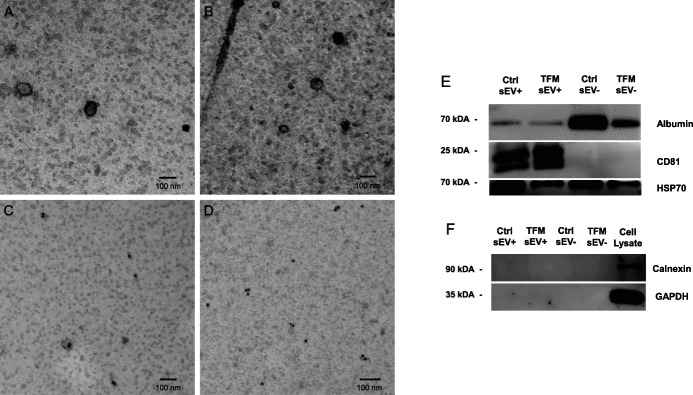
Transmission electron microscopy (TEM) and EV markers of serum-derived sEVs from TFM and control mice. Representative TEM images of sEV morphology and immunogold labeling for sEVs markers. Control (A) and TFM (B) sEVs were fixed and negatively stained with uranyl acetate to assess morphology. Immunogold labeling of control (C) and TFM (D) sEVs with CD81 and CD9 primary antibodies, respectively, and 10 nM gold-labeled secondary antibody after negative staining. Western blots were performed on sEV (sEV+) and sEV deplete (sEV−) fractions collected during sEV isolation for characterization of sEVs markers (5 μg/lane) (E and F). Jurkat cell lysate was used as control for negative markers that are absent in sEV+ or sEV− fractions. sEV, small extracellular vesicle; TEM, transmission electron microscopy; TFM, tibia fracture model.

Serum from TFM and control mice were used to isolate sEVs (sEV+) and sEV deplete (sEV−) fractions. Although the particle concentration was comparable in both groups (Fig. 2A), NTA showed that sEVs of TFM mice were significantly larger than those of control mice (Fig. 2B). The protein content did not differ between the 2 groups (Fig. 2C). Most sEV particles were between the size of 50 to 150 nm in both groups (Fig. 2D). Transmission electron microscopy showed that most particles were intact and <100 nm in size in both control and TFM groups (Figs. 3A–B). We confirmed the presence of sEVs markers by immunogold labeling with CD81 (Fig. 3C) and CD9 (Fig. 3D). Western blotting was used to characterize the depletion of serum-related protein and presence of sEV-related markers.^46^ The serum-related protein albumin was enriched in sEV− fractions; sEV+ fractions were enriched with the classical sEV marker CD81, which was absent in sEV− fractions. Both sEV+ and sEV− fractions ubiquitously expressed HSP70 (Fig. 3E) and lacked the endoplasmic reticulum marker calnexin as well as cytoplasmic marker GAPDH (Fig. 3F).

### 3.3. Cytokine levels in tibia fracture model serum sEV+ and sEV− fractions

To determine the levels of proinflammatory and anti-inflammatory cytokines between control and TFM groups in sEV+ and sEV− fractions, we measured a panel of 23 cytokines. Undetected cytokines were assigned a value of the lower limit of quantitation of the assay for quantitative analysis. When measured solely by concentration, there were no significant changes in any analytes in either sEV+ or sEV− fractions between control or TFM groups (Table 1, supplemental digital content 1, available at http://links.lww.com/PR9/A126, showing nonnormalized cytokines and samples above the lower limit of quantitation). To adjust for sample variation in processing, we normalized assay measurements by their total protein content. Although not significantly different from the control, the sEV+ fraction showed generally decreased cytokine levels in TFM (Table 1A). Several cytokines were significantly increased in the sEV− fraction of TFM mice (Table 1B).

**Table 1:** Cytokine analysis of serum-derived sEVs and small extracellular vesicle–deplete fractions from the tibia fracture model and control mice.

Analyte	sEV fraction						
	Control (n = 6)		TFM (n = 8)		Difference	*P*	*P* Adj
	Mean (pg/mg)	SD	Mean (pg/mg)	SD			
A							
IL-1β	39	13	25	14	−15	0.06	0.38
IL-2	76	54	37	36	−40	0.16	0.38
MIP-1a	70	47	38	24	−32	0.17	0.38
TNFα	63113	22374	44931	25191	−18182	0.18	0.38
IL-5	13808	4924	9853	5524	−3954	0.18	0.38
IL-17	489	174	349	196	−140	0.18	0.38
KC	1134	404	809	454	−325	0.18	0.38
MCP-1	9539	3402	6807	3817	−2732	0.18	0.38
IL-3	1090	576	696	465	−395	0.20	0.38
GM-CSF	406	197	280	142	−126	0.22	0.38
IL-12p40	1826	1585	899	675	−927	0.22	0.38
IL-4	196	133	116	89	−81	0.24	0.38
Eotaxin	1451	704	1023	559	−428	0.25	0.38
IL-9	1343	761	896	540	−446	0.25	0.38
IL-13	36765	47127	12128	8241	−24637	0.26	0.38
G-CSF	2282	1714	1340	975	−942	0.26	0.38
IFNγ	80672	43806	56155	36511	−24516	0.29	0.40
MIP-1β	2743	2317	1561	1557	−1183	0.31	0.40
IL-1α	54	64	25	47	−29	0.37	0.44
IL-10	3634	1492	2824	1956	−810	0.40	0.44
IL-12p70	2725	3173	1328	2548	−1397	0.40	0.44
IL-6	274	197	204	252	−70	0.57	0.60
RANTES	170	226	113	222	−57.03	0.65	0.65

Analyte	sEV-deplete fraction						
	Control (n = 6)		TFM (n = 8)		Difference	*P*	*P* Adj
	Mean (pg/mg)	SD	Mean (pg/mg)	SD			
B							
KC	96	43	189	53	94	**0.00**	**0.02**
IL-13	1623	1202	4148	1533	2524	**0.00**	**0.02**
IL-9	149	56	261	66	111	**0.01**	**0.02**
IL-1β	6	4	13	3	7	**0.01**	**0.02**
Eotaxin	107	22	161	38	54	**0.01**	**0.02**
MCP-1	449	96	693	185	244	**0.01**	**0.02**
GM-CSF	34	9	54	15	20	**0.01**	**0.02**
MIP-1α	7	4	13	4	6	**0.01**	**0.02**
IL-5	993	634	2051	775	1058	**0.02**	**0.03**
IFNγ	4146	1971	8298	4021	4152	**0.03**	**0.04**
IL-2	4	2	7	3	3	**0.03**	**0.04**
IL-6	15	10	26	9	11	0.05	0.06
MIP-1β	76	26	107	28	31	0.05	0.06
IL-10	156	32	208	57	51	0.06	0.06
IL-4	14	8	23	7	8	0.08	0.08
G-CSF	222	104	326	97	104	0.09	0.08
RANTES	8	7	15	7	7	0.09	0.08
TNFα	2186	633	2589	520	403	0.23	0.19
IL-3	34	10	29	9	−5	0.36	0.28
IL-17	19	5	21	5	2	0.49	0.35
IL-1α	1	1	0	0	0	0.49	0.35
IL-12p40	101	23	95	29	−6	0.69	0.46
IL-12p70	61	38	55	25	−6.02	0.74	0.47

Cytokine analysis of serum sEVs **(**A) and sEV deplete fractions (B) from TFM (n = 8) and control (n = 6) mice. Analyte concentration was normalized to protein input. Samples yielding a value < the lower limit of quantification (LLOQ) for each analyte were assigned the LLOQ value for quantitative analysis. Significance was determined by the 2-tailed Welch *t* test, with Benjamini–Hochberg correction for multiple comparisons, *P* value < 0.05 and *P* adj < 0.05 were used as thresholds for significance and FDR, respectively. Significant *P* values are shown in bold.

FDR, false discover rate; LLOQ, lower limit of quantitation; sEV, small extracellular vesicle; TFM, tibia fracture model.

### 3.4. Differentially expressed microRNA in serum-derived small extracellular vesicles from the tibia fracture model

**Figure 4.**
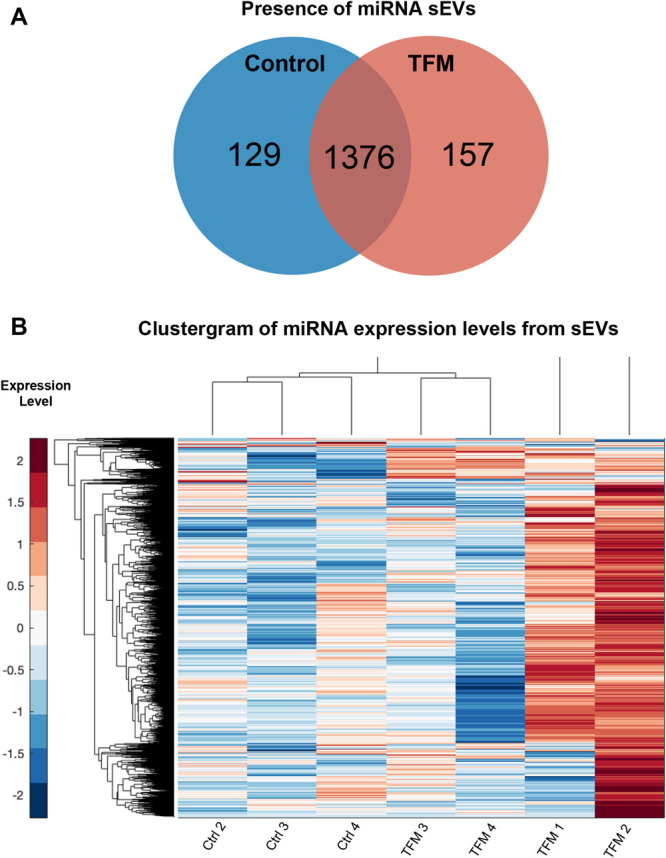
MicroRNA signatures of TFM and control serum-derived sEVs. Venn diagram showing the group expression of miRNAs, where a miRNA was considered present in a group if it had TPM ≥1 in every replicate of that group (A). A hierarchical clustergram of overall miRNA expressions in control and TFM samples, where higher expression is denoted by red and lower by blue and more related terms are closely grouped together (B). miRNA, microRNA; TFM, tibia fracture model; TPM, transcripts per kilobase million.

sEV+ fractions from control and TFM mice were profiled for miRNAs using a whole-transcriptome nuclease protection assay followed by NGS. This method can bypass traditional miRNA isolation methods and directly probe samples for their contents, allowing for smaller sample inputs and decreased variability because of yield and processing methods.^18^ Of the 2,083 miRNA targets probed (Table 2, supplemental digital content 1, available at http://links.lww.com/PR9/A126, showing results of miRNA presence vs, absence analysis), 1,376 were common to both groups with 129 unique to control samples and 157 unique to TFM samples (Fig. 4A). Fifty-seven miRNAs were significantly upregulated in TFM samples compared with controls (Table 2). To better visualize the heterogeneity of the samples, we generated a hierarchical clustergram of the differentially expressed miRNAs using the Pearson correlation coefficient between pairs of samples. Control samples clustered together better than TFM samples, where only 2 TFM samples were clustered separately, whereas the other 2 were similar to control samples (Fig. 4B).

**Table 2:** Differentially expressed microRNA in serum-derived sEVs from the tibia fracture model and control mice.

miRNA	Fold change	*P*	miRNA	Fold change	*P*
miR-148b-3p	2.79	8.33E-05	miR-22-3p	2.55	2.58E-03
miR-222-3p	2.51	1.05E-04	miR-16-5p	2.66	2.82E-03
miR-4711-3p	3.82	1.20E-04	miR-181d-5p	2.81	3.04E-03
let-7a-5p	2.44	1.78E-04	miR-142-5p	2.32	3.36E-03
miR-128-3p	3.24	2.28E-04	miR-484	2.24	3.49E-03
let-7d-5p	2.61	2.77E-04	miR-26a-5p	2.35	3.51E-03
let-7c-5p	2.01	5.24E-04	miR-92a-3p	2.53	3.77E-03
miR-328-3p	2.51	5.54E-04	miR-106b-5p	2.15	3.79E-03
let-7i-5p	2.61	6.14E-04	miR-326	2.02	3.93E-03
miR-191-5p	3.14	6.51E-04	miR-15b-5p	3.01	4.73E-03
miR-31-5p	2.92	7.02E-04	miR-93-5p	2.15	4.85E-03
miR-320a	2.01	8.85E-04	let-7f-5p	2.97	4.91E-03
miR-32-5p	4.76	1.05E-03	miR-29b-3p	2.44	5.06E-03
miR-130b-3p	2.42	1.15E-03	miR-107	2.95	5.58E-03
let-7e-5p	2.52	1.33E-03	miR-19b-3p	2.36	5.60E-03
miR-340-5p	3.12	1.36E-03	miR-548a-5p	6.18	5.74E-03
HK_RPL27	4.06	1.43E-03	miR-27a-3p	2.02	6.38E-03
miR-17-5p	2.13	1.45E-03	miR-185-5p	2.29	6.63E-03
miR-339-5p	2.43	1.50E-03	miR-4649-3p	2.33	6.72E-03
let-7g-5p	2.77	1.56E-03	miR-486-5p	2.01	7.25E-03
miR-744-5p	2.80	1.77E-03	miR-6754-3p	2.55	7.28E-03
miR-15a-5p	2.66	1.81E-03	miR-6754-5p	2.32	7.76E-03
miR-18a-5p	3.03	1.93E-03	miR-106a-5p	2.52	7.80E-03
miR-652-3p	2.26	1.97E-03	miR-6855-3p	4.54	8.06E-03
miR-5089-3p	4.58	2.18E-03	miR-181b-5p	2.66	8.15E-03
miR-130a-3p	2.70	2.19E-03	miR-221-3p	2.03	8.44E-03
miR-4420	4.60	2.22E-03	miR-19a-3p	2.22	8.45E-03
miR-25-3p	2.10	2.39E-03	miR-6778-5p	2.80	9.53E-03
miR-26b-5p	2.14	2.55E-03			

Relative expression levels of significantly altered miRNAs in TFM serum sEVs as compared with control. Expression is reported as fold change relative to an aggregate 13 housekeeping genes. miRNA with a TPM <1 for any sample were removed before analysis. Expression levels were log2 transformed, and geometric means were tested by a 2-tailed *t* test with internal adjustment for the FDR to adjust for multiple comparisons with miRNA that had a *P* value <0.01 and FC > 2 considered to be significant.

FC, fold change; FDR, false discover rate; miRNA, microRNA; TFM, tibia fracture model; TPM, transcripts per kilobase million.

**Figure 5.**
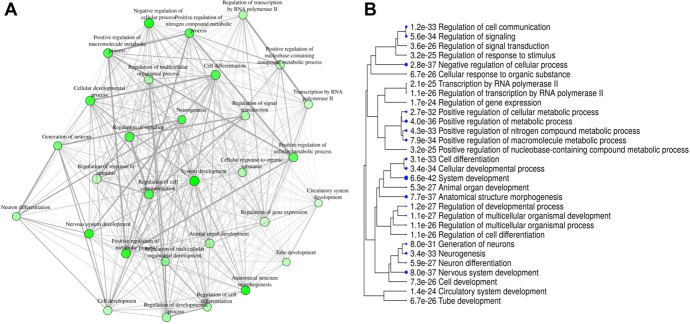
Gene ontology (GO) enrichment analysis of biological processes associated with miRNA target genes. Common target genes of differentially expressed miRNAs in TFM serum sEVs as identified by TargetScan and miRTarbase were used to generate GO enrichment analysis of biological processes of target genes through ShinyGo. A visual network was generated to map biological processes where the size and color of the node represent the number of genes and the FDR of the GO term, respectively, and the thickness of edges between nodes represents the number of genes shared (A). A hierarchical dendrogram of the enriched GO terms where the terms that are most similar are grouped closer together, and the size of the circle represents the FDR (B). FDR, false discover rate; miRNA, microRNA; TFM, tibia fracture model.

To investigate the potential function of miRNA alterations in the serum sEVs of TFM mice, we identified gene targets of differentially expressed miRNAs with TargetScan^1^ and miRTarBase.^13^ Gene targets common to both lists (Table 3, supplemental digital content 1, available at http://links.lww.com/PR9/A126, showing common gene targets of miRNA) were used to generate a GO enrichment analysis of biological processes using the web-based graphical platform ShinyGo.^17^ We produced a visual network and hierarchical tree of the 30 most significant GO terms, allowing a visualization of the biological processes likely to be impacted by the differentially expressed miRNAs. The most enriched processes within the target genes involved cellular and system development, positive metabolic regulation, and neuron-specific programming (Figs. 5A–B). We also generated a list of target genes that belong to all significant GO terms (Table 4, supplemental digital content 1, available at http://links.lww.com/PR9/A126, showing genes associated with each GO term).

**Table 3:** Commonly dysregulated microRNA between the tibia fracture model serum-derived sEVs and patients with complex regional pain syndrome.

Study	McDonald et al. 2014	Douglas et al. 2015 & Orlova et al. 2011	Douglas et al. 2015	Ramanathan et al. 2019
Treatment	Untreated		Ketamine	Plasma exchange
Group comparison	CRPS vs Ctrl		Pre–post treatment	Pre–post treatment
Sample type	Exosome	Whole blood	Whole blood	Exosome
let-7a-5p		X	X	
let-7c-5p		X	X	
let-7d-5p		X	X	
let-7e-5p			X	
let-7f-5p			X	
let-7g-5p		X	X	
miR-15a-5p	X		X	
miR-15b-5p			X	
miR-16-5p		X	X	X
miR-18a-5p		X	X	
miR-19b-3p			X	
miR-22-3p	X			
miR-25-3p		X	X	
miR-26a-5p			X	
miR-26b-5p	X	X	X	X
miR-27a-3p			X	
miR-29b-3p		X	X	
miR-31-5p	X			
miR-92a-3p			X	
miR-93-5p	X	X		
miR-106a-5p			X	
miR-106b-5p		X		
miR-107		X	X	
miR-130b-3p	X	X		
miR-142-5p		X		
miR-148b-3p		X	X	X
miR-185-5p		X		
miR-191-5p		X	X	
miR-222-3p			X	
miR-221-3p		X		
miR-320a		X	X	
miR-339-5p	X		X	
miR-340-5p		X	X	
miR-484		X	X	
miR-486-5p			X	
miR-652-3p		X		
miR-744-5p	X			

Differentially expressed miRNA in TFM-serum–derived sEVs were compared with previously identified miRNAs shown to be associated with CRPS or with CRPS treatment outcomes. Identification of significantly altered miRNAs in previous studies is indicated by X and appropriate column headers indicate sample type, group comparison, treatment, and study.

CRPS, complex regional pain syndrome; miRNA, microRNA; TFM, tibia fracture model.

### 3.5. MicroRNA dysregulated in the tibia fracture model is shared with patients with complex regional pain syndrome

To identify commonly dysregulated miRNAs in sEVs from the serum of TFM and those previously reported in patients with CRPS, we compiled a list of differentially expressed miRNAs from our previous miRNA profiling studies of patients with CRPS. This list contains miRNAs from studies on both serum-derived exosomes^31^ and whole-blood samples.^15,34^ The comparisons thus included miRNAs differentially regulated in patients with CRPS vs controls^34^ and patients with reported miRNA expression changes after plasma exchange^36^ or ketamine treatment^15^ (Table 3). Of the 57 miRNAs dysregulated in TFM–serum-derived sEVs, 30 have been reported as dysregulated in patients with CRPS compared with controls, with 8 in patient exosomes and 23 in whole blood. In addition, 27 TFM miRNAs have been associated with treatment responses to plasma exchange (3 miRNAs in exosomes) and ketamine therapy (27 miRNAs in whole blood). Three specific miRNAs, miR-26b, miR-93, and miR-106b, were commonly dysregulated in both patient exosomes and whole blood. In addition, miR-25, miR-26b, and miR-27 were associated with treatment responses in ketamine therapy.

## 4. Discussion

Small extracellular vesicles harbor selectively packaged RNA and protein from host cells, and their ability to protect cargo from RNAses make sEVs an excellent sample source for miRNA profiling.^12,24^ Here, we profiled sEVs from the serum of male TFM mice to gain insight into miRNA and cytokine dysregulation at the onset of chronic pain because TFM is one of the best models to date to recapitulate the symptoms in patients with CRPS.^6^ Although CRPS shows a higher prevalence in women (3:1),^38^ previous studies reporting miRNA alterations in patients with CRPS include men and women. We chose to evaluate alterations in male mice as an initial study to identify potential biomarkers and targets that may underlie etiology in both sexes.

Although there were no significant differences in sEV concentration and morphology between TFM and control sEVs, the size of TFM sEVs was slightly but significantly larger than controls. This may be simply due to an enhanced distribution of small sEVs within control samples because sEVs contain mixed vesicular populations. Alternatively, smaller EVs may harbor different cargo than larger EVs. Further studies are needed to uncover these potential differences. Previous studies on systemic biomarkers for CRPS have shown limitations in the specificity and utility of soluble cytokines such as TNF-α and IL-6.^4,35,48^ IL-1β,^14^ IL-2, and monocyte chemoattractant protein-1^2,34,48^ have been previously reported as upregulated in patients with CRPS, and MCP-1 is known to play a role in mechanical allodynia in the spinal cord of TFM mice.^16^ Tumor necrosis factor-α, IL-6, and neuropeptide^8^ upregulation is reported in the TFM skin but has not been investigated in the serum. Although we observed significant changes in these and several other cytokines in sEV deplete fractions from TFM mice, there were surprisingly no changes in the cytokine levels of their sEVs. This is consistent with the notion that not all cellular proteins are packaged into sEVs and further supported by our previous study showing that only a subset of proteins are selectively sorted into sEVs in a rodent model of neuropathic pain.^22^ Although some proteins are released directly into circulation, enclosure in sEVs could render protection from proteases. We had previously postulated that circulating sEVs preferentially facilitate long distance communication, whereas free proteins in the serum may contribute to local signaling by a diffusion gradient.^22^

In contrast, there were 57 dysregulated miRNAs in sEVs from TFM as compared with control. Critically, differentially expressed miRNAs in TFM share extensive overlap with miRNAs that are dysregulated in patients with CRPS or associated with patient treatment response (Table 3).We have previously shown the potential utility of miRNA signatures as biomarkers in patients with CRPS by profiling miRNAs from whole blood and serum-derived exosomes.^15,31,34,36^ Although 8 miRNAs showed common dysregulation in sEVs from TFM and patient exosomes, 23 (nearly half) had been previously identified as dysregulated in whole blood from patients with CRPS^34^ and could be due to species difference. Although miRNA changes in whole blood may reflect disease progression, miRNAs exclusively altered in sEVs (miR-15a, miR-22, and miR-31) may provide a more specific and stable signature. We chose to study miRNA dysregulation at the establishment of pain hypersensitivity in TFM (3 weeks) to better understand the miRNA signature associated with this transition. Patient studies included subjects of both sexes with a range of ages, disease durations, and severity whose sustained disease pathology can induce temporal differences^19,42,49^ potentially altering miRNA levels in circulation. Despite these limitations, more than half of the miRNAs associated with the TFM at 3 weeks are both conserved between species and altered in patients with CRPS.

miRNA alterations in TFM sEVs despite the lack of cytokine alterations suggest a more specific role for miRNA. This warrants further investigation of miRNA translation to clinical biomarkers for CRPS. It may be important for future studies of TFM and CRPS to separate the acute and chronic phases to see whether there are temporal changes in miRNA signature as the disease progresses. Previous studies have demonstrated potential roles for differentially expressed miRNAs in patients with CRPS. miR-34a was reported to regulate corticotrophin-releasing hormone receptor 1^39^ and long-noncoding RNA XIST, whereas miR-548d regulates uridine diphosphate-glucuronosyltransferase UGT1A1 mRNA. All the target mRNAs were inversely correlated to specific miRNAs and differentially expressed in whole blood from patients with CRPS and were associated with treatment response.^40^

Unifying the role of miRNA with common mechanisms in TFM and patients with CRPS can help develop translational approaches to treat CRPS. For example, miR-939 has been shown to regulate proinflammatory genes such as IL-6 and nitric oxide synthase, which are often increased in patients with CRPS.^30,37^ Specifically, miR-939 was only increased in B cells isolated from patients with CRPS whole blood, and a functional role of CD20^+^ B cells in the TFM has been demonstrated in mice, suggesting therapies targeting B-cell–mediated pathways may be beneficial.^37^ Although not significant, our data found a change in the miR-939 expression in sEVs of TFM mice (data not shown), suggesting it may still play a mechanistic role in both models. The high similarity of miRNA profiles we observed in TFM mice suggests that there may be common mechanisms associated with TFM development and that of CRPS disease etiology.

Of the dysregulated miRNAs in TFM, miR-25 and miR-27 were directly associated with positive treatment outcomes (Table 5, supplemental digital content 1, available at http://links.lww.com/PR9/A126, expanded table of CRPS-dysregulated miRNA). miR-27 has been shown to be downregulated in mouse models of chronic morphine exposure where analgesic tolerance develops, which may give insight into the ineffectiveness of opioids for CRPS pain management.^44^ Patients with CRPS have impaired bone metabolism,^27^ and responders to ketamine therapy have increased body mass index compared with poor responders.^40^ miR-27 also has key roles in cholesterol synthesis,^25^ bone remodeling,^20,29^ and inflammation.^33^ Taken together, this may suggest a functional role for miR-27 in the regulation of these pathways to mediate recovery in ketamine-responsive patients. miR-25 is a member of the polycistronic miR106b-25 cluster which is one of 3 paralogous miRNA clusters (106b-25, 17-92, and 106a-363). These clusters were strongly represented in TFM mice and patients with CRPS. Although these clusters exhibit a high degree of synergy exemplified by common seed sequences and coregulation,^26^ mechanisms also exist for the regulated transcription of polycistronic miRNAs resulting in cell type–specific expression.^47^ This may explain why only miR-25 is associated with treatment response. A role for miR-25 has been demonstrated in regulating adult neural stem/progenitor cell proliferation^9^ which may be critical for neural plasticity and reorganization in chronic pain disorders such as CRPS. Differences in disease duration as well as packaging in sEVs may explain why miR-25 is increased in TFM sEVS but decreased in long-standing patients with CRPS.

By profiling the miRNA changes in mouse TFM sEVs, we have demonstrated a high degree of similarity to reported miRNA dysregulation of patients with CRPS and broadly conserved pathways of dysregulation. Further exploration of the effects of these miRNAs may yield common mechanisms in disease etiology of TFM and CRPS, allowing for better models and a clearer understanding of how treatment strategies can be developed. Future studies evaluating miRNA dysregulation in female TFM mice will help identify whether miRNA signatures reflect the sex disparity seen in patients with CRPS. It is known that human-specific miRNAs have more targets than miRNAs conserved across species.^3^ Thus, it is crucial that future studies also focus on primate-specific miRNAs and their targets. Despite these limitations, the observed similarities from this study support the case that previously identified miRNAs in patients with CRPS may provide a unique profile that could be used as clinical diagnostic or prognostic biomarkers.

## Disclosures

The authors have no conflicts of interest to declare.

This study was funded by NIH NINDS R01NS102836 to S.K. Ajit and NIH NINDS R01NS117340 and Department of Veterans Affairs I01RX001475 to J.D. Clark. The authors thank Dr. Bradley Nash for critical reading of the article.

## Appendix A. Supplemental digital content

Supplemental digital content associated with this article can be found online at http://links.lww.com/PR9/A126.

## Supplementary Material

SUPPLEMENTARY MATERIAL
